# Properties of Cement Mortar and Ultra-High Strength Concrete Incorporating Graphene Oxide Nanosheets

**DOI:** 10.3390/nano7070187

**Published:** 2017-07-20

**Authors:** Liulei Lu, Dong Ouyang

**Affiliations:** 1 Engineering College, Jiangxi Agricultural University, Nanchang 330045, China; luliulei521@163.com; 2 Research Center of Engineering Materials and Structural Durability, Jinan University, Guangzhou 510632, China; 3 School of Mechanics and Construction Engineering, Jinan University, Guangzhou 510632, China

**Keywords:** cement, graphene oxide, ultra-high strength concrete (UHSC), compressive strength

## Abstract

In this work, the effect of graphene oxide nanosheet (GONS) additives on the properties of cement mortar and ultra-high strength concrete (UHSC) is reported. The resulting GONS-cement composites were easy to prepare and exhibited excellent mechanical properties. However, their fluidity decreased with increasing GONS content. The UHSC specimens were prepared with various amounts of GONSs (0–0.03% by weight of cement). Results indicated that using 0.01% by weight of cement GONSs caused a 7.82% in compressive strength after 28 days of curing. Moreover, adding GONSs improved the flexural strength and deformation ability, with the increase in flexural strength more than that of compressive strength. Furthermore, field-emission scanning electron microscopy (FE-SEM) was used to observe the morphology of the hardened cement paste and UHSC samples. FE-SEM observations showed that the GONSs were well dispersed in the matrix and the bonding of the GONSs and the surrounding cement matrix was strong. Furthermore, FE-SEM observation indicated that the GONSs probably affected the shape of the cement hydration products. However, the growth space for hydrates also had an important effect on the morphology of hydrates. The true hydration mechanism of cement composites with GONSs needs further study.

## 1. Introduction 

Concrete has been extensively used in civil engineering all over the world for more than one hundred years. It has a relatively high compressive strength, but low flexural and tensile strengths. Moreover, cracks are one of the main hidden defects in concrete structures; they cause brittle fractures, shorten the service life, and lower the durability [1]. Generally, the damage and failure of concrete are caused by the nucleation, growth, and coalescence of microcracks [2,3,4]. One strategy to inhibit the formation of cracks is to randomly introduce short, discrete microfibers (e.g., steel fiber or polypropylene fiber) into concrete [5]. However, these microfibers cannot stop or prevent the initiation of microcracks in a concrete matrix. Recently, achievements in nanotechnology have produced some nanofibers with excellent performance, such as carbon nanotubes (CNTs) and graphene nanosheets, which can be used as reinforcements to improve defects of cement-based materials at the nanoscopic level. 

Graphene oxide nanosheets (GONSs), with a unique atom-thick two-dimensional structure, have drawn attention in the past decade due to their large surface and ultra-high strength [6]. GONSs bear hydroxyl and epoxy groups on their basal planes and carbonyl and carboxyl groups on the sheet edges. The presence of these functional groups makes GONSs strongly hydrophilic, which allows GONSs to readily disperse in water [7].The high surface area and unique structure of GONSs may be beneficial for improving bonding strength between graphene sheets and the surrounding cement matrix [8,9,10]. Therefore, GONSs have been accepted as good additives to be incorporated in cement matrix for strengthening. It was reported that GONSs easily formed composites with cement matrix, and effectively increased the mechanical properties of cement pastes or mortars [9,10,11,12,13,14,15,16,17,18,19].

Lv et al. [13] reported that the addition of 0.03% by weight of cement (bwoc) GONSs in cement paste increased the compressive and flexural strengths by 34.3% and 52.4%, respectively. Pan et al. [9] reported that the use of GONSs (0.05% bwoc) also significantly increased the strengths of cement paste. Shang et al. [14] reported that the use of GONSs (0.04% bwoc) increased the compressive strength of cement paste by 15.1%. Horszczaruk et al. [15] reported that the incorporation of GONSs (3% bwoc) into cement resulted in significant enhancement of Young’s modulus. Babak et al. [16] reported that the addition of GONSs (1.5% bwoc) into cement mortar improved the tensile strength by 48%. Gong et al. [17] reported that the introduction of GONSs (0.03% bwoc) into cement paste increased the compressive and tensile strengths by more than 40%. Lu et al. [18] reported that the modification of a magnesium potassium phosphate cement paste with GONSs (0.05% bwoc) improved the compressive and flexural strengths by 6.8% and 8.3%, respectively. Zhou et al. [10] reported that the hybrid GONSs/CNTs (0.02% bwoc GONSs and 0.04% bwoc CNTs) improved the compressive and flexural strengths of cement paste by 23.9% and 16.7%, respectively. Zhao et al. [19] reported that the addition of GONSs (0.022% bwoc) in cement mortar increased the flexural toughness by 33.0%.

Previous studies have focused on the effect of GONSs on the performance of cement pastes or mortars, with the results indicating that the introduction of GONSs in small amounts greatly improved the mechanical properties of cement composites. However, the properties of concrete, especially ultra-high strength concrete (UHSC), has rarely been explored. UHSC is mainly composed of cement, very fine powders such as silica fume and ultra-fine admixtures, aggregates, superplasticizer, and water [20]. UHSC exhibits as very dense microstructures with ultra-high compressive strength exceeding 100 MPa [21,22]. In recent years, the construction industry has shown significant interest in UHSC, with modern high-rise buildings having been constructed using UHSC. However, its safety has been questioned because of possible ultra-brittle failure behavior, and low flexural and tensile strengths [23].

Recently, high-performance CNTs have been used to reinforce concrete by improving these defects at the nanoscopic level. The results of our previous research have shown that CNTs improve the flexural strength and deformation ability of UHSC [1]. Song et al. [24] reported that CNTs lead to large increases in the tensile strength and ultimate strain of the concrete. Similar to CNTs, using GONSs with high specific surface areas might offer another approach to improving UHSC properties. A two-dimensional honeycomb lattice with many oxygen-containing functional groups allows GONSs to disperse more easily in water than CNTs [6]. Notably, a large number of ions, such as Ca^2+^, Fe^3+^, Mg^2+^, and OH^−^, exist in the cement pore solution, which is an alkaline environment [10]. Fan et al. [25] found that GONSs underwent rapid deoxygenation in strong alkali solutions at moderate temperatures. Therefore, Ca(OH)_2_ solution in cement pores had a negative effect on the stability of GONSs. However, superplasticizer used in UHSC mixtures could diminish the effect of Ca(OH)_2_ on GONSs [10,26]. Meanwhile, silica fume used in UHSC could consume Ca(OH)_2_ [27,28], further preventing the reunion of GONSs. Overall, little information is available in the literature concerning the properties of GONS-incorporated UHSC. Therefore, research efforts are clearly needed in this area.

In the present study, the effect of GONSs on the fluidity and mechanical behavior of cement mortar and UHSC was investigated. Moreover, the morphology of hardened cement paste and UHSC samples incorporating GONSs was observed using field-emission scanning electron microscopy (FE-SEM). 

## 2. Experimental Section

### 2.1. Materials

Ordinary Portland cement type II 42.5R (C), silica fume (SF), and ground granulated blast-furnace slag (BS) were used in all mixtures. The chemical analysis and physical properties of C, SF, and BS are listed in Table 1. A polycarboxylate-based superplasticizer (PCs) was used in concrete mixtures for workability purposes. The fine aggregate (FA) used in this study was natural river sand with a fineness modulus of 2.79. The coarse aggregate (CA) was crushed granite with a maximum size of 20 mm. 

The graphene oxide nanosheet (GONS) dispersions used in this study were purchased from Shanxi Institute of Coal Chemistry, China Academy of Sciences (Taiyuan, China), with an oxygen content of 35.91%. They were synthesized by a modified Hummers method [29] and well dispersed in water [30]. The average size and thickness of GONSs are 100–1000 nm and ~0.7 nm [30].

### 2.2. Preparation of Specimens

Three mixtures of cement paste containing GONSs were prepared for FE-SEM observation. The GONSs were added in the amount of 0%, 0.05%, and 0.25% (bwoc), and the water/cement ratio of the cement paste was 0.5. The cement paste was analyzed after 28 days of standard curing (Relative humidity: ≥95%, Temperature: 20 ± 1 °C) in a standard curing box.

Six mixtures of cement mortar were prepared by 450 g cement, 1350 g ISO standard sand, 225 g water, and a certain amount of GONSs. GONSs were added at levels of 0.00%, 0.01%, 0.03%, 0.05%, 0.08%, and 0.10% bwoc in the mortar mixtures. According to the GB/T17671-1999 standard [31], the mixtures were molded into a cuboid of 40 mm × 40 mm × 160 mm to test the flexural and compressive strengths.

Three mixtures of the ultra-high strength concrete (UHSC) were prepared by mixing cementitious materials (cm), aggregates, GONS dispersions, water, and PCs. The ratio of water-to-cementitious materials (w/cm) was maintained as 0.20 for all the concrete admixtures. GONSs were added at levels of 0.00%, 0.01%, and 0.03% bwoc in the UHSC mixtures, which are numbered as UGO00, UGO01, and UGO03, respectively. The mixture proportions for the UHSC are shown in Table 2. The water in the GONS dispersions and PCs solution should be deducted in the mixing water. The detailed preparation process is described in our previous work [1]. After four minutes of being prepared, the mixtures were poured into oiled molds and compacted on a vibration table. Then, the specimens were covered with a plastic sheet for 24 h. All the specimens were cured in a standard curing room (Relative humidity: ≥95%, Temperature: 20 ± 2 °C) until the specified testing age.

### 2.3. Test Methods

#### 2.3.1. Characterization of GONSs

The Fourier transform infrared (FTIR) spectra of the GONS and pristine graphite samples were recorded in the range of 4000–500 cm^−1^ using an Equinox 55 FTIR spectrometer (Bruker, Karlsruhe, Germany) with a resolution of 4 cm^−1^ for 32 scans.

The X-ray diffraction (XRD) patterns of the GONS and graphite powders were measured in a 2*θ* range of 5–50° with CuKα radiation (λ = 0.154056 nm) using a MiniFlex 600 X-ray diffractmeter (Riguku, Tokyo, Japan) working at an accelerating voltage of 40 kV and current of 15 mA. Afterwards, the diffractograms were refined by the Rietveld method to gain a quantitative phase analysis.

The field-emission transmission electron microscopy (FE-TEM) micrographs of the GONS samples were obtained using a JEM-2100F instrument (Jeol, Tokyo, Japan) working at 200 kV.

#### 2.3.2. Fluidity Measurements

The mini-slump flow test was carried out to measure the fluidity of cement mortars. After mortar mixing, the fluidity was evaluated by measuring the mini-slump flow using the standard procedures of the GB/T2419-2005 standard [32].

To investigate the effect of GONSs on the fluidity of the UHSC samples, the slump and slump flow tests were carried out. After mixing, fresh concrete mixtures were poured into a slump cone (top diameter, 100 mm; bottom diameter, 200 mm; height, 300 mm). The testing procedures followed the GB/T50080-2002 standard [33]. 

#### 2.3.3. Flexural and Compressive Strength Tests

To evaluate the effect of GONSs on the mechanical properties of the UHSC samples, flexural and compressive strength tests were conducted on the cuboids of 100 mm × 100 mm × 300 mm with a loading rate of 20 kN/min and cubes of 100 mm × 100 mm × 100 mm with a loading rate of 1.0 MPa/s according to the GB/T50081-2002 standard [34], respectively. Each test was conducted in triplicate. The flexural strength was calculated using the following formula:

(1) $$f=\frac{3FL}{2bh^{2}}$$

where f is the flexural strength (MPa), F is the failure load (N), *L* is the span between two supporting point (200 mm), and *b* and *h* are the width and height of the specimens (mm), respectively.

#### 2.3.4. Morphology Observation

The cement paste and UHSC samples with a size of approximately 5 mm × 5 mm × 5 mm were prepared at 28 days after being crushed. The samples were kept in alcohol until the SEM observation. The field-emission scanning electron microscopy (FE-SEM) micrographs of the samples were recorded using a ULTRA 55 instrument (Carl Zeiss, Oberkochen, Germany) operating at 5.0 kV, or a Nova NANOSEM 430 instrument (FEI, Hillsboro, OR, USA) working at 10.0 kV.

## 3. Results and Discussion

### 3.1. Characterization of Graphene Oxide Nanosheets 

**Figure 1:**
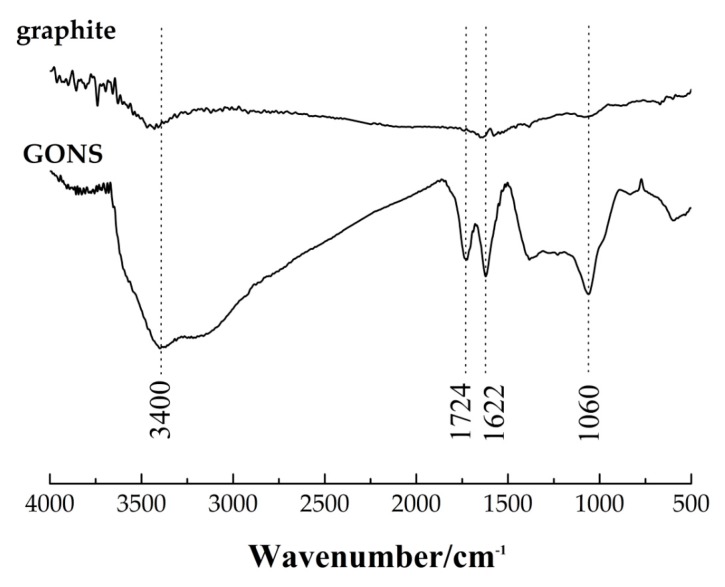
FTIR spectra of graphene oxide nanosheet (GONS) and graphite.

The FTIR spectra of graphene oxide nanosheet (GONS) and graphite are shown in Figure 1. For GONS, the peak at 3400 cm^−1^ shows the presence of stretching vibration of hydroxyl O-H. The characteristic peaks at 1724 cm^−1^ and 1622 cm^−1^ respectively appear for carboxyl C=O and aromatic C=C, the peak at 1060 cm^−1^ shows the presence of epoxy C-O [35,36]. In comparison with graphite, these results demonstrated that the oxygen functional groups such as -OH, -COOH, and -O- were introduced onto GONS structure through the oxidation of graphite.

**Figure 2:**
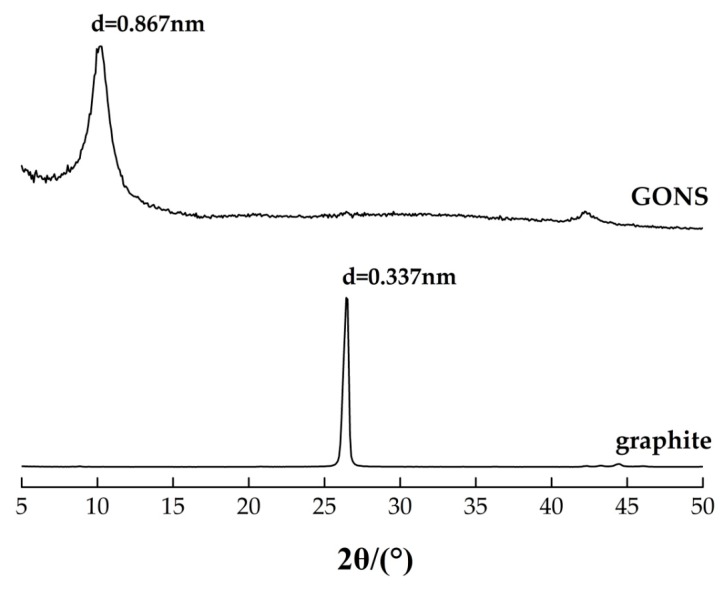
XRD patterns of GONS and graphite.

The XRD patterns of GONS and graphite are plotted in Figure 2. The results indicated that the interlayer distance of GONS had expanded to 0.867 nm compared with that of graphite, 0.337 nm. The results confirmed that the oxygen functional groups had penetrated into the graphite interlayer and weakened the interaction between the layers, which help to disperse GONS into aqueous solutions easily and form a stable nanosheets suspension [13]. 

**Figure 3:**
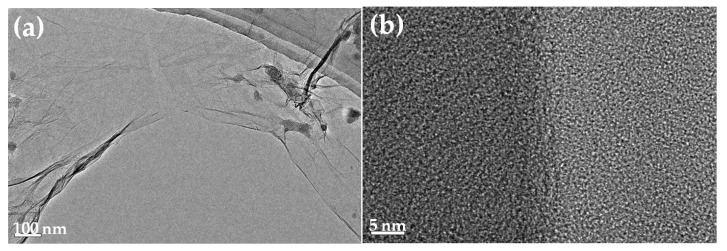
TEM images of GONSs: (**a**) the observed ridge or crease demonstrating a wrinkled surface texture of GONSs; and (**b**) the observed 'honeycomb' exhibiting a graphene network structure.

The TEM images of GONSs are shown in Figure 3. Transparent sheets with a large number of dark ripples were observed using low-magnification TEM (Figure 3a). The observed ridge or crease demonstrated a wrinkled surface texture of GONSs [9]. The transparency revealed that the sheets contained GONSs of only a few layers [37]. Figure 3b shows that the GONSs exhibited a graphene network structure.

### 3.2. Fluidity

**Figure 4:**
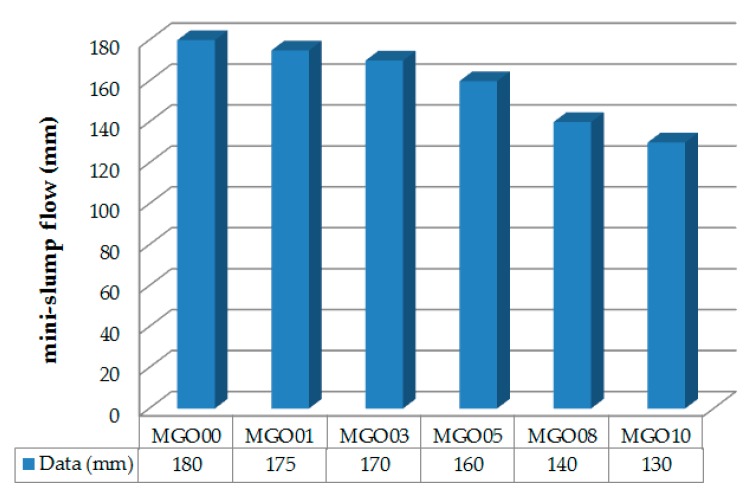
Effect of GONSs contents on the fluidity of cement mortars. The mortar samples containing 0.00%, 0.01%, 0.03%, 0.05%, 0.08%, and 0.10% by weight of cement (bwoc) GONSs are numbered as MGO00, MGO01, MGO03, MGO05, MGO08, and MGO10, respectively.

The results of the mini-slump flow tests are shown in Figure 4. The GONS content had a large influence on the fluidity of the mortar mixtures. The mini-slump flow of cement mortar without GONSs was 180 mm. When 0.10% by weight of cement (bwoc) GONSs was added, the mini-slump flow was 130 mm, which was 27.8% lower than that of the mortar without GONSs. The mini-slump flow decreased with increasing GONS content, indicating that GONS additives reduced the fluidity of the cement mortars.

The results of the slump and slump flow tests are showed in Table 3. The results showed that the fluidity of ultra-high strength concrete (UHSC) samples was reduced by increasing the GONS content. The slump and slump flow of UHSC without GONSs were approximately 240 mm and 450 mm, respectively. When 0.03% bwoc GONSs was added, the slump and slump flow were 220 mm and 380 mm, representing reductions of 8.3% and 15.6%, respectively. The decrease in amplitude of the slump flow was greater than that of the slump, indicating that the incorporation of GONSs increased the viscidity of UHSC.

These results indicated that GONS additives decreased the fluidity of cement composites. A similar conclusion can also be found in the literatures [9,14], which reported that the addition of GONSs reduced the fluidity of fresh cement paste. These results might be due to the large surface area of GONSs, which decreases the available water in fresh mixture from wetting GONSs [9].

### 3.3. Mechanical Properties

**Figure 5:**
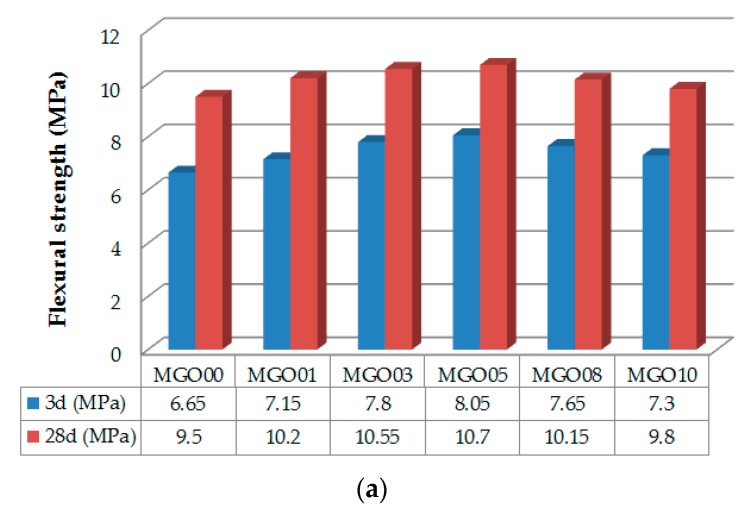

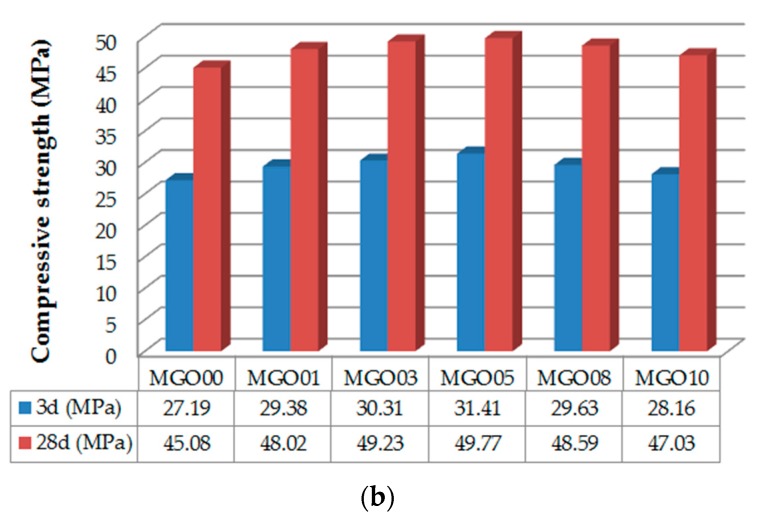
(**a**) Flexural strength and (**b**) compressive strength of GONSs-incorporated mortars.

The results of the strength tests for cement mortars are showed in Figure 5. The strength increased with increasing GONS content until it reached 0.05% bwoc, followed by a decrease in strength with a further increase in GONS content to 0.10% bwoc. The flexural and compressive strengths of the specimens without GONSs (MGO00) were 6.65 MPa and 27.19 MPa at three days, and 9.5 MPa and 45.08 MPa at 28 days, respectively. Figure 5a shows that specimens containing 0.05% bwoc GONSs (MGO05) exhibited a 21.1% increase in flexural strength and 15.5% increase in compressive strength at three days, compared with MGO00. Moreover, Figure 5b shows that the flexural and compressive strengths of MGO05 at 28 days had increased by 12.6% and 10.4% compared with those of MGO00, respectively. Therefore, the optimum GONS content was clearly 0.05% bwoc. However, the strengths of the specimens containing 0.03% bwoc GONSs (MGO03) were slightly lower than those of MGO05, especially at 28 days. Previous reports concluded that small amounts of GONSs (approx. 0.01–0.05 wt %) provide good improvements in the mechanical properties of cement composites [10,11,12,13,14].

The results of the strength tests for the UHSC samples are listed in Table 3. The compressive strength of the specimen without GONSs (UGO00) was 117.34 MPa after 28 days of curing. With GONSs contents of 0.01% bwoc (UGO01) and 0.03% bwoc (UGO03), the compressive strength of the specimens were 126.52 MPa and 122.73 MPa, representing 7.82% and 4.59% increases compared with UGO00, respectively. Notably, the compressive strength was measured using non-standard specimens, meaning that the results should be reduced by a strength conversion coefficient of 0.95 [22,23]. The compressive strengths of the concrete specimens prepared in this study all exceeded 100 MPa, which meets the technical requirements of UHSC. After seven days of curing, UGO01 and UGO03 showed 3.66% and 4.55% increases in compressive strength, and 11.88% and 6.95% increases in flexural strength compared with UGO00, respectively. These different changing trends in flexural and compressive strength might be due to experimental error. An apparent increase in strength was observed when incorporating small amounts of GONSs into UHSC, and the optimum GONS content was 0.01% bwoc. 

**Figure 6:**
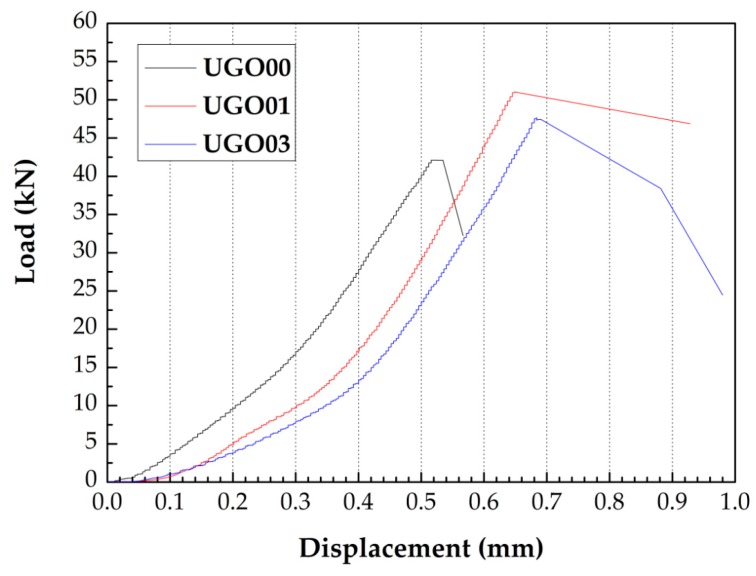
Load-displacement curves of GONSs-incorporated UHSC.

Figure 6 shows the typical flexural stress-strain curves of the UHSC containing GONSs. The addition of GONSs significantly increased the deformation ability of UHSC. The failure displacement increased with increasing GONS content.

The above results indicated that GONS additives improved the flexural and compressive strengths of cement composites, with the increase in compressive strength less than that of flexural strength. Moreover, GONS additives improved the deformation ability of UHSC.

### 3.4. Micrograph

**Figure 7:**
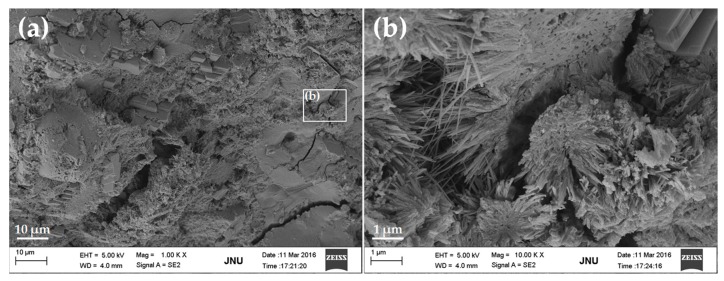
SEM images of fracture surface of plain cement paste: (**a**) typical hydration morphology at low magnification; and (**b**) the local amplification for the area in the white box marked in image (**a**). Images were taken using a Carl Zeiss ULTRA 55 instrument operating at 5.0 kV.

SEM images of the fracture surface of plain cement paste (without GONSs) after curing for 28 days are shown in Figure 7. Figure 7a shows that hydration products are mainly composed of needle-like ettringite (AFt), laminated Ca(OH)_2_, and flocculent C-S-H gel, etc. C-S-H and AFt can be clearly seen in the pore (Figure 7b).

**Figure 8:**
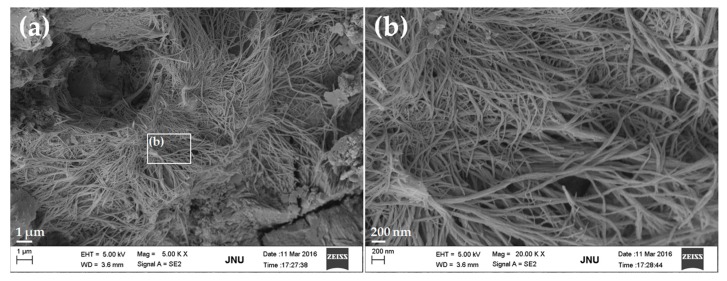

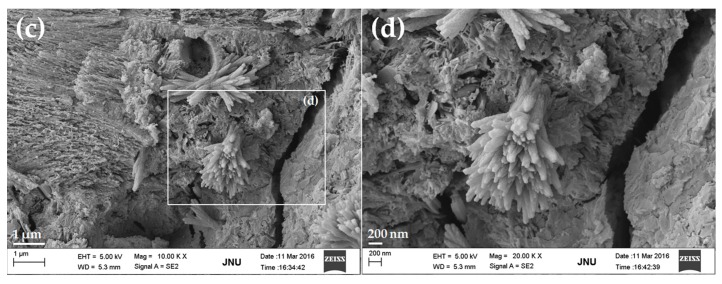
SEM images of fibrous-like and flower-like crystals in the cement paste with 0.25% bwoc GONSs: (**a**) a cluster of fibrous crystals found in the pore; (**b**) the local amplification for the area with the white box marked in image (**a**); (**c**) some flower-like crystals observed on the surface; and (**d**) the local amplification for the area with white box marked in the image (**c**).

Figure 8 shows SEM images of the fracture surface of cement paste containing 0.25% bwoc GONSs after curing for 28 days. The morphology of hydration products was mostly similar to that of plain cement paste in most areas. However, a cluster of fibrous-like crystals emerged in the pore (Figure 8a,b), which were very different from the needle-like AFt (Figure 7b). Figure 8b shows the local amplification of Figure 8a. A detailed examination of Figure 8b showed that the fibrous-like crystals present were 20–40 nm in size. These fibrous-like crystals might be beneficial for improving the flexural strength. Moreover, some flower-like crystals can also be found on the surface of harden cement paste (Figure 8c,d), with morphologies similar to those of cement hydration crystals previously reported [13,38].

**Figure 9:**
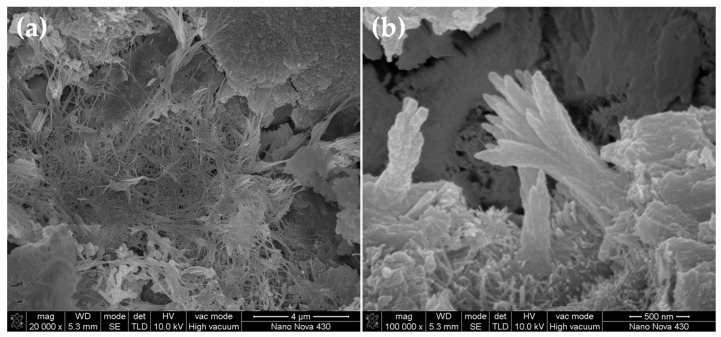
SEM images of fibrous-like and flower-like crystals in the cement paste with 0.05% bwoc GONSs: (**a**) a cluster of fibrous crystals and (**b**) some flower-like crystals found in the pore. Images were taken using a FEI Nova NANOSEM 430 instrument operating at 10.0 kV.

Figure 9 shows SEM images of the fracture surface of cement paste containing 0.05% bwoc GONSs after curing for 28 days. These images were recorded using a Nova NANOSEM 430 instrument working at 10.0 kV. The fibrous-like and flower-like crystals shown in Figure 8 were observed again, and confirmed to be C-S-H and Ca(OH)_2_ by Energy Dispersive Spectroscopy (Table 4), respectively. These results indicated that GONSs might have an effect on the shape of the cement hydration products.

**Figure 10:**
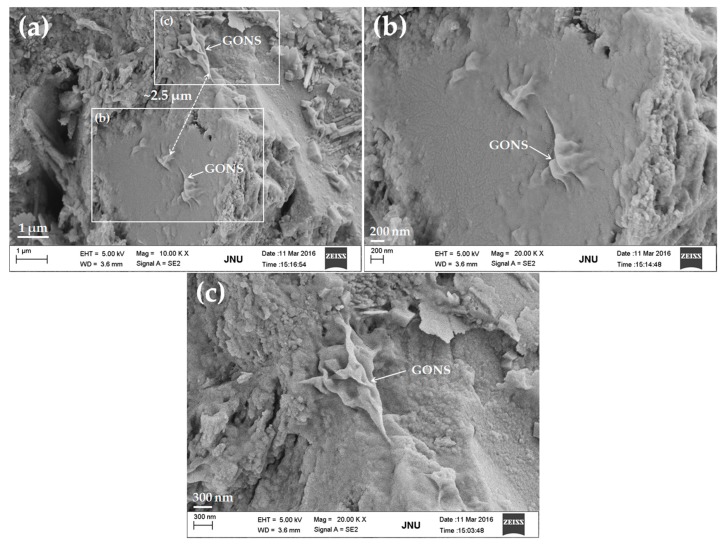
SEM images of GONSs in the GONS-incorporated UHSC sample: (**a**) Some existing GONSs individually observed on the surface; (**b**) the local amplification for one GONS in the white box marked in image (**a**); and (**c**) the local amplification for another GONS in the white box marked in image (**a**).

Figure 10 shows SEM images of the UHSC sample mixed with 0.03% bwoc GONSs after curing for 28 days. At a low weight fraction of GONSs, it was rather challenging to identify the GONSs by SEM analysis due to its planar geometry and the hydration products coating on the GONSs [9]. Fortunately, the GONSs were found in the cement matrix, as shown in Figure 10a. The distance between two GONSs was ~2.5 μm, and no GONS aggregate was observed, suggesting that the GONSs were well dispersed in the matrix, with each GONS existing individually. Recently, Li et al. [39,40] reported that GONS aggregates formed in cement paste due to the chemical cross-linking of divalent calcium cations [41], which were abundant in cement composites. However, with the addition of silica fume, the dispersion of GONSs was greatly improved [39]. Silica fume is a very reactive pozzolan, with average particle size (~100 nm) approximately 100 times finer than that of Portland cement particles. There are two possible reasons for the prevention of GONS aggregations by silica fume [28,39]. Firstly, fine silica fume particles could prevent aggregation of GONSs by mechanically separating GONS from calcium ions. Secondly, silica fume reacted with Ca(OH)_2_ to produce C-S-H and reduced the concentration of calcium ions around the GONSs [39]. Moreover, the superplasticizer used in cement composites could also relieve the negative effect of calcium cations on GONS [10,26]. The reason might be the steric effect of the large molecules (poly-carboxylate superplasticizer) adsorbed on the GONSs, which prevented the reunion of GONSs [10]. In this study, both silica fume and poly-carboxylate superplasticizer were used to prepare the GONS-incorporated UHSC mixtures. Therefore, GONSs could be effectively dispersed in the UHSC matrix. Figure 10b shows the local amplification of Figure 10a. A detailed examination of Figure 10b showed that GONS was securely anchored within the surface of the matrix microstructure, which indicated that the bonding of GONSs with the surrounding cement matrix was strong. This was attributed to the reaction between the C-S-H and oxygen functional groups on the surface of the GONSs [8,9]. As a result, the mechanical properties of cement composites were improved, as mentioned in Section 3.3. Figure 10b,c showed that GONS tended to twist and interlace with cement hydrates, which was not conducive to give full play to its excellent performance.

Notably, due to the dense microstructure, the different cement hydration crystals shown in Figure 8 were not observed on the surface of the UHSC sample with GONSs. It was concluded that the growth space significantly affected the morphology of the hydrates. The hydrates can achieve ‘free growth’ in unlimited space, whereas the hydrates’ growth is restrained if the space is small and compact. The space of hydrate growth depends on the sample preparation method for hardened cement paste [42]. There is no clear evidence that the presence of GONSs affected the morphology of hydrates, as reported previously [13,38]. Therefore, further study is needed to collect more meaningful statistics about the effect of GONSs on the shapes of cement hydration crystals. 

It should be noted that a possible reinforcing mechanism of GONSs on UHSC was caused by crack resistance [9,16,40]. Pan et al. [9] reported that GONSs could effectively deflect, or force, cracks to tilt and twist around the sheets. However, the crack bridging and pulling out, commonly observed in cement composites incorporating carbon nanotubes [1,8], were very difficult to be found in the GONS-incorporated cement composites due to a low GONS volume fraction and its planar geometry. Notably, the properties of hardened cement paste were very close to those of ceramics. Thus, the related studies of ceramic matrix composites lent support to the above point of view. Xia et al. [43] reported that the fracture toughness of ceramic composites increased due to crack bridging and pulling out of the reduced graphene oxide. Liu et al. [44] reported that fracture propagation in ceramic composites was resisted by bridging, pullout, and two-dimensional deflection of graphene platelets. However, research in this area is very inadequate, which also calls for further study in the future.

## 4. Conclusions

The following conclusions have, hence, been drawn:

1. The fluidity of cement mortar and ultra-high strength concrete (UHSC) decreased with the increasing addition of graphene oxide nanosheets (GONSs).
2. Adding GONSs improved the flexural and compressive strengths of cement mortar and UHSC, with the increase in flexural strength more than that of compressive strength. Particularly, the compressive strength of UHSC incorporating 0.01% by weight of cement GONSs after curing for 28 days increased by 7.82% than that of UHSC without GONSs (117.34 MPa). Moreover, GONS additives significantly increased the deformation ability of UHSC.
3. FE-SEM observations showed that GONSs were well dispersed in the cement matrix and the bonding of GONSs with the surrounding matrix was strong.
4. The microstructural studies indicated that GONSs might have an effect on the shape of the cement hydration products. However, the growth space for hydrates also had an important effect on the morphology of hydrates. Therefore, further study is needed to collect more meaningful statistics about the effect of GONSs on cement hydration mechanisms.
5. Research concerning about the crack resistance mechanism of GONSs on cement composites is still very inadequate, which also calls for further study in the future.

## Figures and Tables

**Table 1:** Chemical composition and physical properties of cement (C), silica fume (SF), and ground granulated blast-furnace slag (BS). LOI, SG, and SSA are the abbreviation of loss on ignition, specific gravity, and specific surface area, respectively.

Material	Chemical Composition (wt %)							Physical Properties	
	SiO_2_	Al_2_O_3_	Fe_2_O_3_	CaO	MgO	SO_3_	LOI	SG	SSA (m^2^/kg)
C	20.13	4.53	4.11	63.88	1.35	2.28	2.82	3.10	331
SF	93.85	0.69	0.17	0.75	1.22	0.41	1.88	2.20	~20,000
BS	44.91	14.86	–	31.08	7.18	0.65	1.80	2.83	1228

**Table 2:** Mixture proportions of ultra-high strength concrete incorporating graphene oxide nanosheets (GONSs). C, cement; SF, silica fume; BS, ground granulated blast-furnace slag; FA, fine aggregate; CA, coarse aggregate; W, water; PCs, polycarboxylate-based superplasticizer.

NO.	w/cm	GONS (wt %)	Quantities (kg/m^3^)							
			C	SF	BS	GONS	FA	CA	W	PCs
UGO00	0.2	0.00	420	60	120	0.000	798	976	120	15
UGO01	0.2	0.01	420	60	120	0.042	798	976	120	15
UGO03	0.2	0.03	420	60	120	0.126	798	976	120	15

**Table 3:** Fluidity, flexural strength, and compressive strength of ultra-high strength concrete (UHSC) with different content of GONSs. The UHSC samples containing 0.00%, 0.01%, and 0.03% bwoc GONSs are numbered as UGO00, UGO01, and UGO03, respectively.

No.	Fluidity (mm)		Flexural Strength (MPa)	Compressive Strength (MPa)	
	Slump	Slump Flow	7 d	7 d	28 d
UGO00	240	450	8.92 (0.00)	90.60 (0.00)	117.34 (0.00)
UGO01	235	420	9.98 (11.88)	93.92 (3.66)	126.52 (7.82)
UGO03	220	380	9.54 (6.95)	94.73 (4.55)	122.73 (4.59)

**Table 4:** The chemical composites of cement hydration crystals.

Cement Hydration Crystals	Element Percentage (%)				Hydration Products
	Ca	Si	O	C	
Figure 9a: fibrous-like crystals	20.59	12.91	52.07	14.43	C-S-H
Figure 9b: flower-like crystals	32.73	-	54.84	12.42	Ca(OH)_2_
